# Kohonen Artificial Neural Network and Multivariate Analysis in the Identification of Proteome Changes during Early and Long Aging of Bovine *Longissimus dorsi* Muscle Using SWATH Mass Spectrometry

**DOI:** 10.1021/acs.jafc.1c03578

**Published:** 2021-09-15

**Authors:** Jessica Brandi, Elisa Robotti, Marcello Manfredi, Elettra Barberis, Emilio Marengo, Enrico Novelli, Daniela Cecconi

**Affiliations:** † Department of Biotechnology, University of Verona, Strada le Grazie 15, Verona 37134, Italy; ‡ Department of Sciences and Technological Innovation, University of Piemonte Orientale, Alessandria 15121, Italy; § Department of Translational Medicine and Center for Translational Research on Autoimmune Diseases, University of Piemonte Orientale, Novara 28100, Italy; ∥ Department of Comparative Biomedicine and Food Science, University of Padua, Legnaro, Padua 35122, Italy; ⊥ Department of Translational Medicine, University of Piemonte Orientale, Novara 28100, Italy

**Keywords:** *longissimus dorsi*, chemometric techniques, supervised Kohonen networks, PLS-DA, SWATH-MS

## Abstract

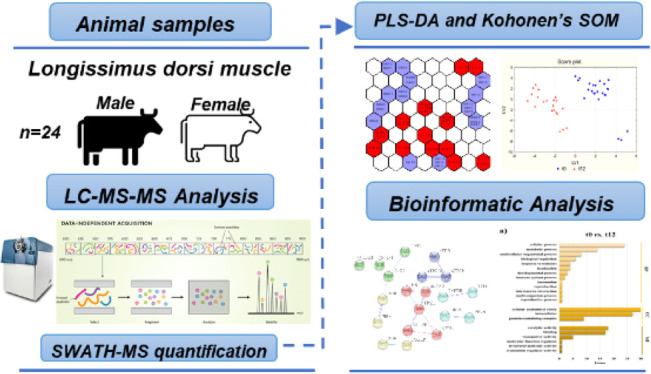

To study proteomic changes involved in tenderization of *Longissimus dorsi*, Charolais heifers and bulls muscles were sampled after early and long aging (12 or 26 days). Sensory evaluation and instrumental tenderness measurement were performed. Proteins were analyzed by gel-free proteomics. By pattern recognition (principal component analysis and Kohonen’s self-organizing maps) and classification (partial least squares-discriminant analysis) tools, 58 and 86 dysregulated proteins were detected after 12 and 26 days of aging, respectively. Tenderness was positively correlated mainly with metabolic enzymes (PYGM, PGAM2, TPI1, PGK1, and PFKM) and negatively with keratins. Downregulation in hemoglobin subunits and carbonic anhydrase 3 levels was relevant after 12 days of aging, while mimecan and collagen chains levels were reduced after 26 days of aging. Bioinformatics indicated that aging involves a prevalence of metabolic pathways after late and long periods. These findings provide a deeper understanding of changes involved in aging of beef and indicate a powerful method for future proteomics studies.

## Introduction

Bovine muscle tenderness represents the first quality required by the consumer, and it is the result of complex biological processes which occur in the striated muscles and in the closely linked tissues during aging. The aging process is an extended time of storage beyond the resolution of rigor and plays a key role in enhancing meat palatability and quality parameters. Despite interest from meat scientists, the biochemical changes underling meat tenderness are not yet fully clarified. Nevertheless, there are some major points of agreement about meat tenderizing processes, which include, among others, proteolysis, as well as cellular stress reactions.^1^

Over the last decade, proteomics has been applied to identify biomarkers useful to predict the tenderness of bovine meat.^2−8^ A meta-proteomics analysis of 12 different gel-based proteomics studies that identified biomarkers from *Longissimus thoracis* (LT) and *Semitendinosus* (ST) muscles, of different types of cattle from beef, hardy or mixed breeds, revealed strong dissimilarities to identify generic biomarkers of beef tenderness.^9^ Indeed, muscle and gender specificities were identified. Particularly, structural and contractile proteins, proteins involved in protection against oxidative stress and apoptosis, as well as proteins of energy metabolism, 70 family HSPs, and proteasome subunits were more involved in LT than in ST tenderness. Moreover, another recent meta-proteomics analysis of the same research group,^10^ which included 28 proteomics experiments investigating beef tenderness of solely *Longissimus* muscle [i.e., *Longissimus dorsi* (LD), *Longissimus lumborum*, and LT] detected a panel of 33 proteins. This panel, which warrants further validation as universal biomarkers of beef Longissimus tenderness, included proteins from the muscle structure and contraction pathway, energy metabolism, response to stress, as well as oxidative stress proteins and one protein involved in cell detoxification. Interestingly, 16 proteins belonging to this panel (i.e., ACTA1, MYH1, MYL1, TNNT3, TNNI2, CKM, ENO3, ENO1, GAPDH, PGM1, PKM, HSPB1, HSPB6, CRYAB, PARK7, and CA3) were also identified in our previous gel-based proteomics study^2^ as related to tenderness of the LD muscle in Charolais cattle. Here, we hypothesized that a high throughput gel-free proteomics approach and advanced statistical analyses would lead to an increased detection of biomarkers of LD tenderness in Charolais beef breed.

In the present study, to broaden our understanding about the biological mechanisms underpinning tenderization of LD of Charolais cattle and to update the current status of protein biomarker discovery involved in beef tenderness, a label-free quantitative proteomics analysis based on Sequential Window Acquisition of All Theoretical Mass Spectra (SWATH-MS) coupled to multivariate statistical analysis and advanced data mining was performed. In particular, pattern recognition tools were applied: principal component analysis (PCA) and Kohonen’s networks, the latter of which is able to identify complex relationships between the variables based on nonlinear mapping. Then, partial least squares-discriminant analysis (PLS-DA) was applied as a classification method, coupled to a variable selection procedure in backward elimination, to identify the biomarkers of tenderization. These multivariate analyses allowed for an exhaustive detection of biomarkers and contemporarily focused on the achievement of models with the highest predictive ability.

## Materials and Methods

All chemicals described in this section were purchased from Sigma-Aldrich (Milan, Italy).

### Animals and Meat

The study was conducted on female (*n* = 4) and male (*n* = 4) Charolais breed animals 14–18 and 15–19 months of age, respectively, belonging to commercial batches intended for human consumption. They were born in France and fattened in Italy for the last 6 months in two farms that adopted the same feeding plan. All the animals were slaughtered the same day at an industrial abattoir (Reg. EC/853/2004) that was around 1 h away by truck. After 2 h of resting time in the lairage, the animals were captive-bold stunned and dressed (Reg. EC/853/2004) and submitted to official inspection (Reg EC/854/2004), and after that, the carcasses were classified (Reg. EC/1308/2013, Reg. EC/1182/2017, and Reg. EC/1184/2017). The mean weight of the carcasses was 338.3 ± 28.5 kg for females and 421.6 ± 15.6 kg for males, whereas the EC classification (category, carcass conformation, and fat cover) was EU3 for females and AU2 for males. A snippet of the LD muscle 30 min post-mortem was cut between the fifth and sixth dorsal vertebra, immediately frozen in liquid nitrogen, then stored at −80 °C, and was used as the sample of time 0 (t0, control). The carcasses were then suspended from the Achilles tendon, kept at 12 °C for 2 h, and then placed in a chilled room (0–2 °C). Muscle pH and temperature were recorded at 40 min post-mortem and each hour for the next six in the LD muscle between fifth and sixth vertebra using a Xerolyt Plus penetration electrode (Mettler Toledo, Urdof, Switzerland) assembled on a portable pH meter (Knick 911, Berlin, Germany) and a food core thermometer Testo 106 (Testo AG, Lenzkirch, Germany). The pH was then measured at 24 h post-mortem. 4 days later, the sample of LD was excided from the right side of each carcass (last eight dorsal vertebras). Each striploin bone-in was shared in two parts (two blocks of four vertebrae each) and individually vacuum-packaged into a shrink barrier bag (polyamide/polyethylene, 140 μm total thickness) used for commercial purposes. The samples were transferred to the laboratory and stored in darkness at 2–4 °C until their use. From the side of the sixth vertebrae of each animal, before packaging, two pieces of meat (around 5 g) were taken, singularly vacuum-packaged, and stored together with the blocks of bone meat for 12 (t12) and 26 (t26) days when they were unpacked, immediately frozen in liquid nitrogen, added with protease inhibitors, and placed at −80 °C for proteomics analysis.

### Sampling and Cooking Procedures

At 12- and 26-days post-mortem, half of the samples stored under vacuum were deboned and sliced into several steaks of 25 mm thickness. The first six slices, from the cranial side, were used for sensory analysis and two more slices for instrumental tenderness measurement after cooking. Cooking was done using a clam shell grill with a ribbed surface on top and bottom (Sirman CORT RR PS, Marsango, PD, Italy) set at 250 ± 5 °C, as checked by an infrared thermometer (Testo 831, Testo AG, Lenzkirch, Germany), up to an internal temperature of 63 ± 0.5 °C monitored by a type T thermocouple (Testo 108, Testo AG, Lenzkirch, Germany). Steaks for texture analysis were immediately chilled to 4 °C using a blast chiller (Tecnodom AT05ISO, Vigodarzere, PD, Italy), then wrapped with aluminum foil, and stored at 2 ± 1 °C overnight before coring.^11^ The steaks for sensory evaluation were immediately submitted to test.

### Sensory Evaluation and Warner–Bratzler Shear Force

A quantitative descriptive sensory analysis was conducted by a 10-member panel (6 men and 4 women, with ages ranging from 26 to 55 years) according to ISO 8586:2012 who had skills with sensory analysis (ISO 13299:2016) on different kinds of foods. The cooked slices still warm were freed of removable epimysia connective tissue and reduced in cubes of 1.27 cm side by means of a sample sizer, and two cubes for an assessor, taken from different sites of the slices, were served for three sessions a day over three consecutive days (three or two samples/session). In each day, the samples were presented to assessors in a randomized order. Samples were evaluated on a 10-point scale (1 = the least intense and 10 = the most intense) for odor, taste, flavor, juiciness, sweet, tenderness, saltiness, bitterness, acidity, gumminess, and overall acceptability. The sessions were held far from breakfast and/or lunch, and samples were tested at intervals of about 5 min. The tests were done in a laboratory, where the temperature was set at 20 °C (ISO 8589:2007).

Instrumental texture was measured using the Warner–Bratzler shear (WBS) test.^12^ Six to eight strips, 1.27 cores, from each cooked steak were cut parallel to the longitudinal orientation of the muscle fibers. The cores were sheared using a TA-HDi texture analyzer (Stable Micro Systems, Survey, UK). The cutting blade was 2 mm thick and had a speed of 200 mm/min when cutting through strips.^13^ The results were expressed as the maximum shear force in kg/cm^2^.

### Protein Extraction and Proteomics Analysis

Pieces of 200 mg of frozen muscle tissue were homogenized as previously reported^2^ in 1.5 mL of lysis/solubilizing solution containing 7 M urea, 2 M thiourea, 3% CHAPS, 20 mM Tris, and 1X inhibitor cocktail tablet (Complete Mini; Roche) by using nitrogen liquid. Proteins were then precipitated overnight at −20 °C in four volumes of cold acetone, pelleted by centrifugation at 14,000*x g* for 15 min at 4 °C, and resuspended in 600 μL of 100 mM NH_4_HCO_3_. After protein quantification by using BCA protein assay, proteins were digested and peptides were subjected to SWATH-MS analysis, as previously described.^14^ Briefly, liquid chromatography tandem mass spectroscopy analyses were performed using a micro-LC Eksigent Technologies system (Dublin, USA) interfaced with a 5600+ TripleTOF instrument (AB Sciex, Concord, Canada). The injection volume of each sample was 4.0 μL. Samples used to generate the SWATH spectral library were subjected to data-dependent acquisition (DDA) and then to cyclic data-independent analysis using a 25 Da window. The MS data were acquired by using Analyst TF v.1.7 (AB Sciex). PeakView v.1.2.0.3 and Protein Pilot v.4.2 (AB Sciex) software were used to generate the peak list. The MS files were searched for protein identification using Protein Pilot and Mascot (Matrix Science, Inc., Boston, MA). Quantification was performed with PeakView and MarkerView v.1.2 (AB Sciex) by integrating the extracted ion chromatogram of all the unique ions for a given peptide. SwathXtend was employed to build an integrated assay library with the DDA acquisitions using a protein FDR threshold of 1%. The six peptides per protein with the highest MS^1^ intensity and six transitions per peptide were extracted from the SWATH files. Peptides with FDR lower than 1% were exported for the univariate and multivariate statistical analyses.

### Statistical Analyses

The data of WBS were analyzed by the analysis of variance (ANOVA) test with animal, days of aging, and their interaction as the main effects. When the ANOVA was significant, means were compared using the Tukey b posteriori test. The statistically significant difference was established at *p* < 0.05.

The differences in protein expression between samples were analyzed statistically by Student’s t-test using a *p*-value <0.05 and fold change (FC) >1.3 and <0.769. The multivariate statistical analysis was carried out using PCA after autoscaling^15^ for a preliminary exploration of the data set. The study was focused to identify candidate biomarkers in four different comparisons: t0 vs t12 (biomarkers of early maturation); t12 vs t26 (biomarkers of late maturation); t0 vs t26 (biomarkers of long maturation); and t0 vs t12&t26 (general biomarkers of maturation). Kohonen’s self-organizing maps (SOMs) were also applied to each of the comparisons performed.^16^ Kohonen’s SOMs are artificial neural networks, that is, mathematical algorithms able to solve complex problems by simulating the human brain functioning. Kohonen’s SOMs are based on an auto-associative unsupervised algorithm: the data described by their multivariate structure (here, each sample described by its protein profile) are presented to the network, which groups them depending on their similarity. This similarity can also be local, that is, related to a subset of the variables employed to describe the problem. Kohonen’s SOMs are based on a single layer of neurons, usually arranged in a square (top layer), where the samples appear grouped at the end of the learning phase. Below each neuron of the top layer, there is a column of cells, one cell for each descriptor (X variables, here the proteins), which contains the weights of the network. During one epoch of the learning process, each sample is presented in turn to the network. For each sample, the distance between the sample and every column of weights is calculated. The column with the minimum distance is considered as the winning neuron. The weights of this neuron are modified so that, at the subsequent cycle, the distance of the same sample from the winning neuron is the smallest. A similar correction is applied to the neurons in the neighborhood of the winner. This correction decreases with the distance and usually also decreases with the number of epochs. In the beginning, all the network is affected by the corrections, while in the last cycles, only the winning neuron is corrected. Similarly, in the beginning, the learning rate, that is, the amount of correction introduced, is larger than in the last cycles. The aim of Kohonen learning is to map similar signals to similar neuron positions. The final result is a map of neurons, where the most similar samples are in the same cell or in close cells. The weights below each neuron give insights into the reason for the clusterization of the objects. In the present work, for all the comparisons, Kohonen’s networks were run with the following settings: toroidal boundary, batch algorithm, hexagonal topology, random initialization of weights, and learning rate decreasing linearly from 0.5 to 0.01; for the general maturation (t0 vs t12&t26 samples), a top map of 10 × 10 neurons and 300 training epochs provided the best results, while a top map with 8 × 8 neurons and 200 training epochs were adopted for the other comparisons.

PLS-DA^17,15^ was then applied to identify candidate biomarkers in each of the four performed comparisons, with a variable selection strategy in backward elimination, allowing the selection of the most discriminant variables according to the smallest percentage classification error rate in cross-validation (leave-more-out procedure with 5 cancellation groups of 20% of the samples randomly selected each time, procedure repeated 1000 times): at each iteration of the variable selection algorithm, the variable with the lowest VIP score was eliminated.^18^ The final results were reported both in fitting and in cross-validation and compared to the PLS-DA models calculated with the variables selected on the basis of the monovariate approach. The classification performances were evaluated on the basis of several parameters: accuracy %, non-error-rate %, sensitivity, specificity, and precision.^19^

PCA and PLS-DA were carried out by MATLAB R2014a (The Mathworks, Natick, MA, USA) using in-house-developed routines and the Classification Toolbox from Milano Chemometrics;^20^ Kohonen SOMs were built with the Kohonen and CPANN toolbox for MATLAB from Milano Chemometrics.^16^ Graphical representations were carried out by MATLAB, Statistica v.7 (Statsoft Inc., Tulsa, OK, USA), and Excel 2016 (Microsoft Corporation, Redmond, WA, USA).

### Bioinformatics Analyses

Gene ontology (GO) annotations of the identified proteins were screened against *Bos Taurus* database using the PANTHER platform v.15.0 (http://www.pantherdb.org/).^21^ Protein–protein interactions and enriched KEGG pathways were detected using STRING tool v.11.0 (http://string-db.org) setting *p* < 0.05 and gene count >2 as the cut-off point and *Bos Taurus* as taxonomy.^22^ Interactions were retrieved at the high confidence level (score 0.7) based on experimental and database knowledge, excluding all the other prediction methods implemented in STRING (such as co-expression and text mining). Proteins were subjected to k-means clustering for five clusters, and disconnected nodes were excluded.

## Results and Discussion

### Sensory and Tenderness Evaluation of Early and Long Aging of LD Muscles

**Figure 1:**
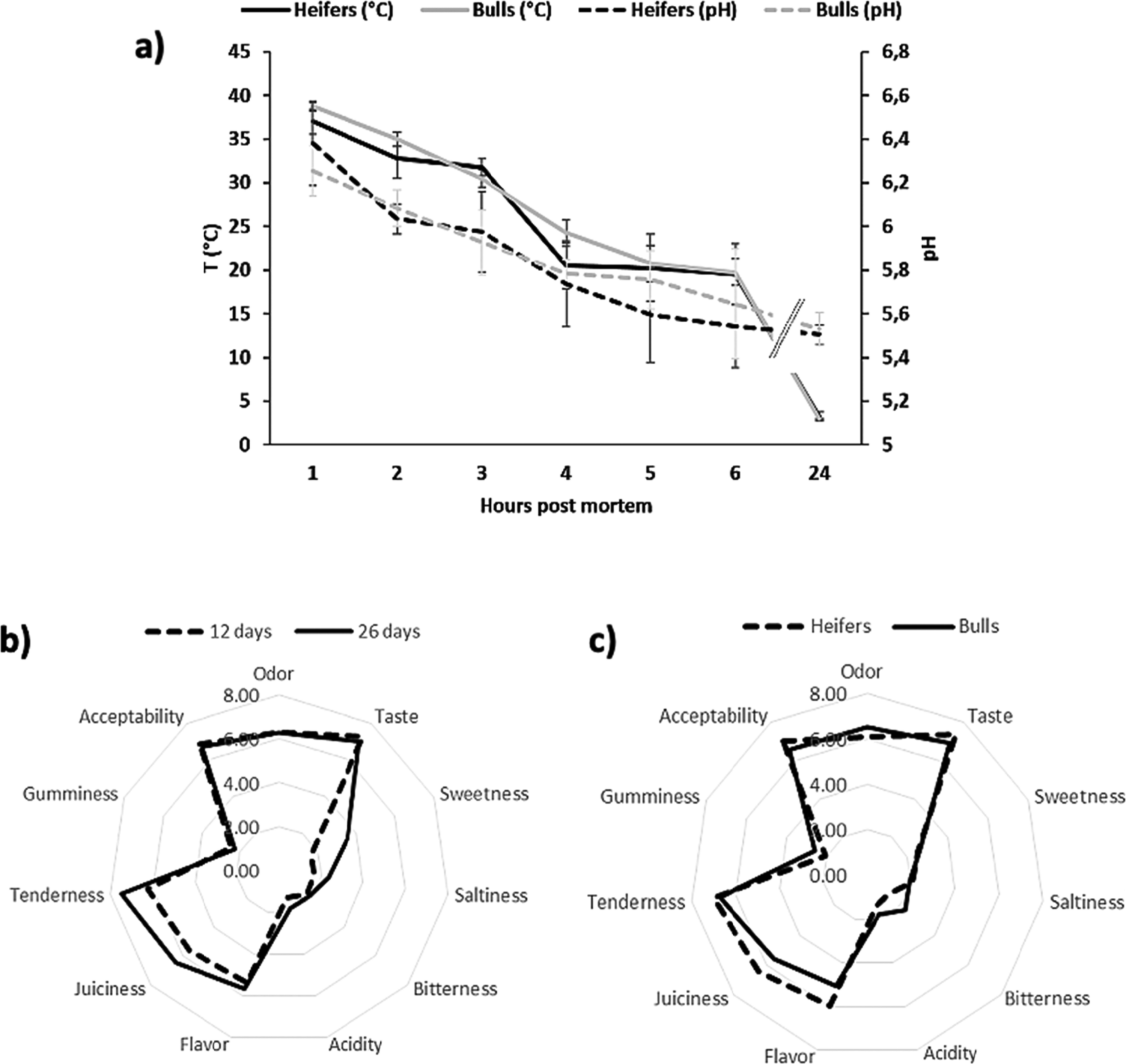
Kinetic of post-mortem pH decline and beef sensory quality. (a) pH values and temperature of LD muscles of heifers and bulls in the first 6 h post-mortem (mean ± standard deviation). The pooled ultimate pH value (24 h p.m.) was 5.51 ± 0.04 and 5.53 ± 0.07 (*p* > 0.05) for heifers and bulls respectively. (b) Sensory attribute analysis of LD muscle after early (D12) and long (D26) aging time. (c) Sensory attribute analysis of LD muscle comparing heifers and bulls.

Sensory quality traits, tenderness, and proteomic changes of the bovine LD muscle during early and long maturation time were analyzed, following the experimental design reported in Figure S1. The kinetics of pH decline measured in the LD muscle in the first 6 h of post-mortem is shown in Figure 1a. The decrease appeared relatively linear and without significant differences between males and females. However, the mean slope of the pH curve of heifers was significantly greater (*p* < 0.05) than that of bulls. The pH measured after 1- and 2-h post-mortem, which was always higher than 6 for all animals, except one female, was significantly correlated (*p* < 0.05) with the shear force measured at 26 days of aging (*r* = −0.723 and −0.709 respectively, *p* < 0.05). Therefore, it seems that the higher the pH in the first hour’s post-mortem, the lower the shear force after 26 days of aging. It has been demonstrated that the temperature of m. *L. dorsi* at pH 6 (approximately) is on average equal to 32 and 35 °C in heifers and bulls, respectively.^23^ Here, the results showed that the average temperature at pH 6 was lower, around 25 °C, which could be related to the lengthening of the aging time from 12 to 26 days, which caused the significant variation of some sensory attributes. The intensity of sweet, salty (*p* < 0.001), and sour (*p* < 0.01) tastes was increased, similar to the juiciness (*p* < 0.01) and tenderness (*p* < 0.001) in the samples aged for 26 days compared to those at 12 days (Figure 1b). Several studies have demonstrated the relationship between sensory tenderness and aging: in particular, an increase in sensory tenderness of the *L. dorsi* muscle has been shown, which was correlated with the aging times between 7 and 56 days,^24^ from 9 to 14 days of aging,^25^ and finally between 3 and 35 days of aging.^26^

Regarding the comparison between bulls and heifers, significant differences were observed (Figure 1c): the intensity of odor (*p* < 0.05), bitterness (*p* < 0.001), acidity (*p* < 0.05), and gumminess (*p* < 0.005) was greater in bulls than in heifers, while the intensity of taste (*p* < 0.05), flavor (*p* < 0.001), juiciness (*p* < 0.001), and acceptability (*p* < 0.01) was higher in heifers than in bulls. The meat of the heifers showed a higher sensory quality than that of the bulls, as highlighted also by other authors.^27,28^ The juiciness was correlated with tenderness (*r* = 0.756, *p* < 0.01) and with gumminess (*r* = −0.643, *p* < 0.01), while acceptability was correlated with gumminess (*r* = −0.567, *p* < 0.05). The shear force measurements showed a significant effect of the aging period, with an average difference of −11.15 N between 26 and 12 days (Table 1). Indeed, the shear force values were significantly correlated with sensory tenderness (*r* = −0.664, *p* < 0.01). Nonetheless, the aging time acted differently on bulls and heifers. At 12 days, the WBS was significantly greater in heifers than in bulls (39.7 vs 31.7, *p* < 0.01), while at 26 days, the values were comparable (24.8 vs 24.3, *p* > 0.05). It is interesting to note the correlation between the shear force and the time lapse: a recent study has observed the decrease in the shear force values at 14 days of aging compared to the measurements conducted at 9 days on m. *L. dorsi*.^25^ It has been found that shear force values at 14 days were lower than those measured at 7 days^29^ and that shear force values at 4 weeks of aging were significantly lower than those measured after 2 weeks.^30^

**Table 1:** Instrumental Measurements of LD Muscle Tenderness after 12 and 26 Days of Aging^a^

	DWBS	pooled std. dev.	*F*	*p*-value
Heifer 1 (*n*_D12_ = 5; *n*_D26_ = 5)	–10.6	6.6		
Heifer 2 (*n*_D12_ = 5; *n*_D26_ = 5)	–23	14.4		
Heifer 3 (*n*_D12_ = 5; *n*_D26_ = 5)	–13.8	13.6		
Heifer 4 (*n*_D12_ = 5; *n*_D26_ = 5)	–12.2	7.4		
Bull 1 (*n*_D12_ = 5; *n*_D26_ = 5)	–6.8	6.9		
Bull 2 (*n*_D12_ = 5; *n*_D26_ = 5)	–0.8	8.0		
Bull 3 (*n*_D12_ = 5; *n*_D26_ = 5)	–12.4	9.0		
Bull 4 (*n*_D12_ = 5; *n*_D26_ = 5)	–9.6	10.9		
Average DWBS	–11.15			
Animal			2.88	<0.05
Days			39.49	<0.001
Animal x Days			1.59	ns

a Difference of WBS force values (DWBS) calculated as the difference between the mean WBS at t26 and at t12 for each sample (animal) independently, the corresponding standard deviation (Pooled Std. Dev.) calculated by pooling the standard deviations obtained for each animal independently, as well as *F* values and significance of animal and aging days’ effects and their interaction on WBS data, are reported.

### Proteome Changes in the Bovine LD Muscle during Early and Long Aging

Proteins from muscle samples of Charolais heifers and bulls (0, 12, and 26 days after slaughter) were subjected to SWATH-MS analysis (Table S1). Each biological replicate was measured in three technical replicates, and one sample (sample code 10/1, female at t12) was discarded for technical problems. Figure S2 reports the results of PCA applied to the overall data set consisting of 69 samples (24 t0 samples, 21 t12 samples, and 24 t26 samples) described by 137 variables (protein signals) after autoscaling. The first two PCs explained about 34% of the overall information (PC_1_: 21.03% and PC_2_: 13.77%): the samples were quite well grouped according to the maturation time, but no evident information on the gender was present. However, the differences in the protein profile related to gender were investigated also by monovariate statistics. Student’s *t* test (*p* < 0.05, FC > 1.3 and <0.79) detected five less abundant (HSP90AA1, TF, S100A1, MYOZ2, and PABPC1) and four more abundant (FBP2, UQCRC1, ERCC6L, and PGM1) proteins in meat of heifers with respect to meat of bulls, regardless of the state of maturation. These differences can be explained by the hormonal influence on muscle proteins, which have already been demonstrated.^31^ Then, univariate and multivariate statistical analyses were carried out in order to identify dysregulated proteins related to meat aging.

The proteomic profile was then correlated to the sensory evaluation and the WBS force measurements by means of the correlation matrix, as reported in Table S1. The correlation matrix reports the proteins identified on the rows and the sensory variables and the WBS force on the columns; in each position of the matrix, the correlation between the two variables indicated on the corresponding row and column is reported: correlation values range between −1 (corresponding to two variables with a negative linear dependence) and 1 (corresponding to two variables with a positive linear relationship), while values around 0 are reported for variables with no linear dependence. Because the correlation is an evaluation of the linearity between two variables, if it is around 0, the two variables could be completely independent or show a nonlinear behavior. The red values in the correlation matrix indicate correlations statistically significant at *p* < 0.05. In general, a few proteins can be identified as positively or negatively correlated to the sensorial evaluations or the WBS force. In particular, among the eight proteins detected as positively correlated with tenderness, there were ATP2A1, which plays a role in apoptotic cell death,^32^ ACTN3 and CKM muscle proteins, as well as five different metabolic enzymes (PYGM, PGAM2, TPI1, PGK1, and PFKM). Fourteen proteins negatively correlate with tenderness, eight of which were cytoskeletal proteins, that is, keratins.

#### Early-Aging Biomarkers

**Figure 2:**
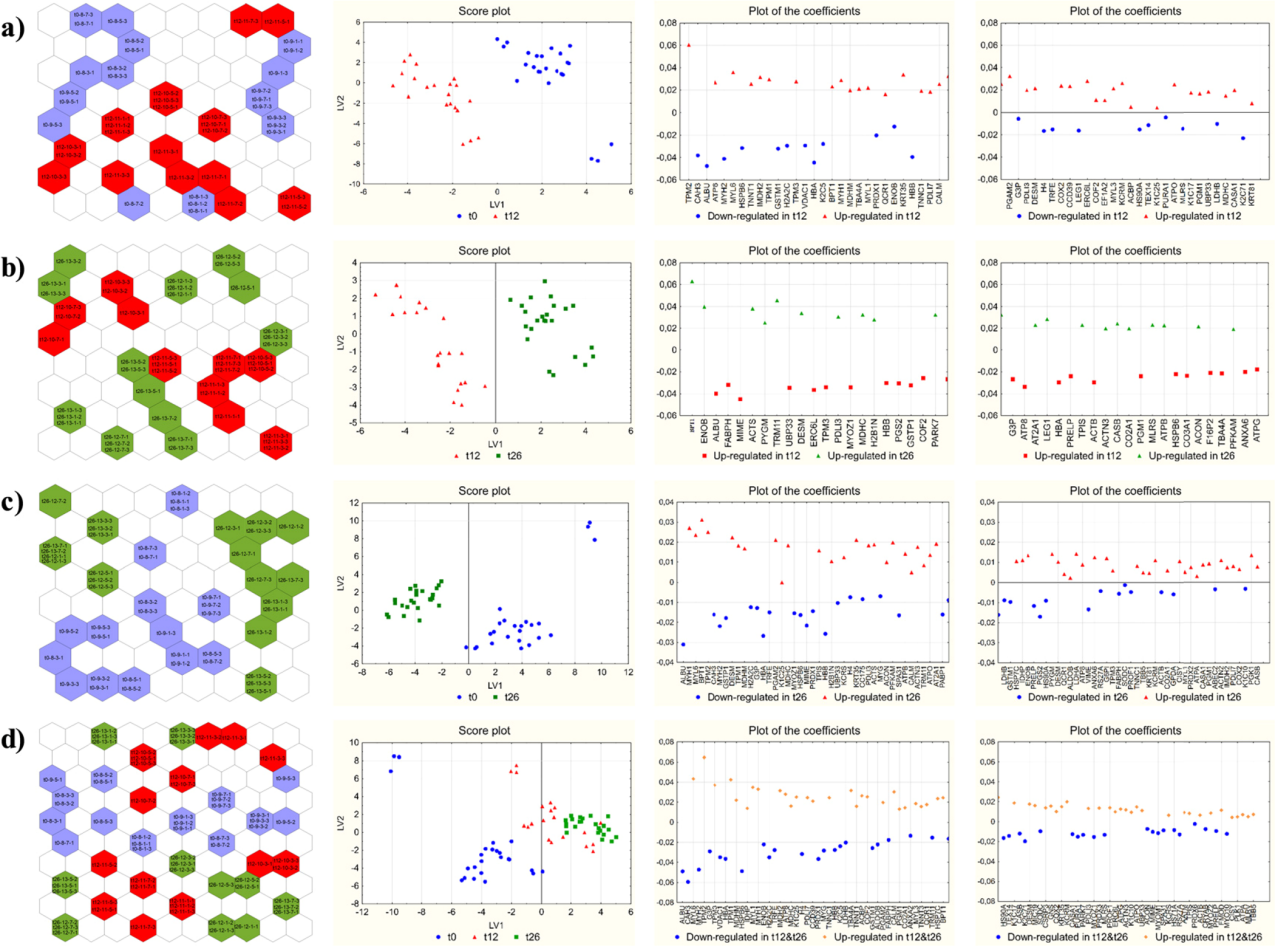
Results of multivariate statistics. Kohonen’s top map, score plot of the first two LVs and plot of the coefficients for each PLS-DA model calculated: (a) early-aging biomarkers (58 variables in the final model); (b) late-aging biomarkers (43 variables in the final model); (c) long-aging biomarkers (86 variables in the final model); (d) biomarkers of general aging (97 variables in the final model). The plots of the coefficients were separated in two panels for more clarity.

Kohonen’s SOMs were applied to the early-maturation data set, consisting of 45 samples (24 samples at t0 and 21 at t12) described by 137 variables. The top map (Figure 2a) shows the samples well separated in the two groups: similar samples are located by the algorithm in the same neuron or in adjacent neurons. Due to the toroidal structure, the neurons located on the boundary at the extreme left and right show similar characteristics, and the same applies to neurons located on the boundary at the bottom or at the top of the map. In the top map, the three replications of each sample are in the same neuron or in adjacent neurons, showing that the instrumental variability is smaller than the biological one. PCA was then applied to the weights calculated for each neuron: the score plot and the loading plot are reported in Figure S3 (panel a). The score plot reports the neurons of the top map in the space given by the first two PCs: neurons where samples of the two classes are present are indicated with different labels, while empty neurons are indicated as black circles. PCA highlights a good separation of the neurons where different groups of samples are located: t0 samples mainly at positive scores on PC_2_ and t12 samples in the opposite position. The loading plot shows a group of variables with larger signals in t12 samples at positive loadings on PC_1_ and negative ones on PC_2_ and another group at negative loadings on PC_1_ and positive on PC_2_, with the opposite behavior.

PLS-DA coupled to the variable selection algorithm selected 58 variables and 2 latent variables (LVs), providing the correct classification of 100% of the samples in fitting and almost the same result in cross-validation (accuracy = 99.77%, 20% of the samples in the test set at each iteration, 1000 iterations) (Table S1). As comparison, the model including all the variables provided the best results in cross-validation (20% of the samples in the test set at each iteration, 1000 iterations) with 7 LVs, achieving an accuracy of 97% (100% in fitting): the variable selection procedure was therefore effective in the identification of the most discriminating variables, achieving a simpler model with higher performances in cross-validation and reducing the risk of overfitting. The first two LVs explained 34.35% of the total variance of the X variables and 45.73% of the class memberships (details are given in Table S1). The score plot of the first two LVs and the plot of the coefficients of the model calculated with 2 LVs are reported in Figure 2a: the samples appeared very well separated, with t12 samples at negative values on LV_1_ and the controls at positive values; the plot of the coefficients showed at positive values the 36 proteins upregulated at t12, and at negative values the 22 proteins downregulated at t12.

**Figure 3:**
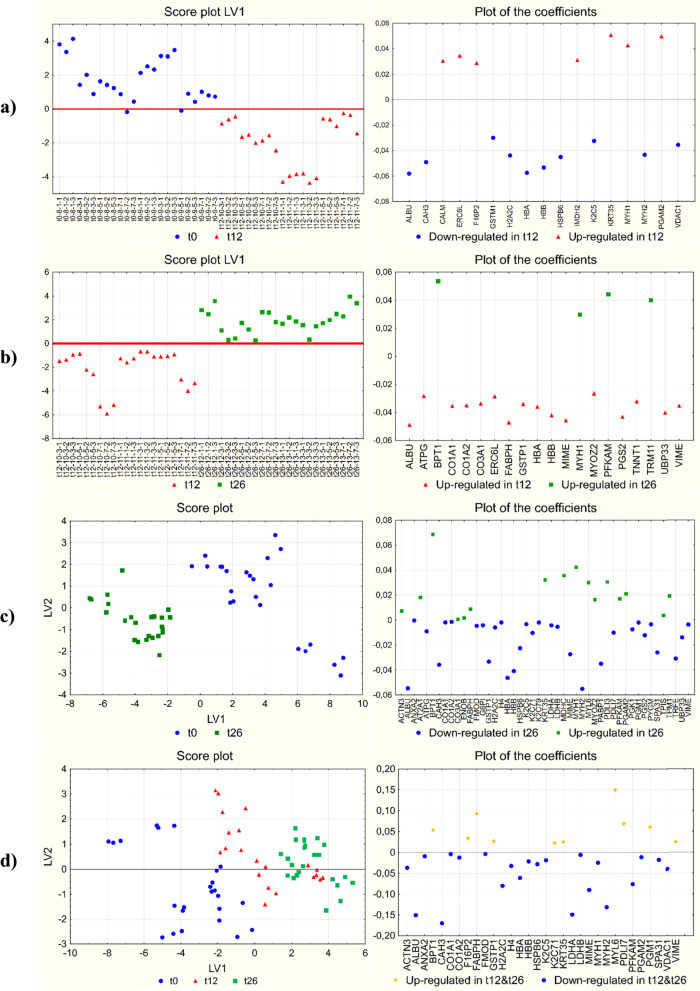
Results of monovariate statistics. Score plot of the first two LVs and plot of the coefficients for each PLS-DA model calculated: (a) early-aging biomarkers (17 variables in the final model); (b) late-aging biomarkers (20 variables in the final model); (c) long-aging biomarkers (47 variables in the final model); (d) biomarkers of general aging (32 variables in the final model).

The monovariate approach highlighted 17 proteins as deregulated (*p* < 0.05) in the comparison between controls and 12 days aged meat (Table S1). These biomarkers were used as input to a PLS-DA model: the best predictive results were obtained with one LV in the final model (accuracy = 95.56% in fitting and 95.39% in cross-validation; Table S1). The corresponding score and coefficient plots are reported in Figure 3a: the samples appeared quite well separated along LV_1_, with two control samples misclassified in the t12 class (the solid horizontal line corresponds to the delimiter between the two classes). The plot of the coefficients showed 7 proteins as upregulated at t12 and 10 proteins as downregulated after 12 days.

#### Late-Aging Biomarkers

Kohonen’s SOMs were applied to the late-maturation data set, consisting of 45 samples (21 samples at t12 and 24 samples at t26) described by 137 variables. The top map (Figure 2b) shows the samples well separated in the two groups, considering its toroidal structure. As for early aging, the three replications of each sample are in the same neuron or in adjacent neurons of the top map, showing that the instrumental variability is smaller than the biological one. PCA, applied to the weights calculated for each neuron, gave the results reported in Figure S3 (panel b); also in this case, neurons containing t26 or t12 samples were indicated by a different marker, while neurons containing no samples were indicated as a black circle. In general, most of neurons containing t26 samples appear to be located at negative scores on PC_1_ and at positive ones on PC_2_, while most of neurons containing t12 samples are at positive scores on PC_1_. The corresponding loading plot highlights a group of variables at negative loadings on PC_1_: among these, those with a positive score on PC_2_ have larger signals in t26 samples, while those with a negative loading on PC_2_ have a larger signal on the three t12 samples at negative scores on PC_2_; the variables with the most positive loadings on PC_1_ show instead larger signals in t12 samples.

PLS-DA was then applied; in this case, 43 variables were included in the final model by the variable selection algorithm, providing the best prediction results with 2 LVs: the perfect classification of all the samples in fitting and almost perfect classification (accuracy = 99.98%) in cross-validation (20% of the samples in the test set at each iteration, 1000 iterations) (Table S1). Also in this case, the model including all the variables showed the best results in cross-validation (20% of the samples in the test set at each iteration, 1000 iterations) with 6 LVs, with an accuracy of 96% (100% in fitting). As for the previous case, the variable selection procedure proved to be effective in the identification of the most discriminating biomarkers, providing a simpler model and higher performances in cross-validation and reducing the risk of overfitting. The first two LVs explained 27.95% of the total variance of the X variables and 46.25% of class belonging (Table S1). The score plot of the first two LVs and the corresponding plot of the coefficients are reported in Figure 2b: the samples appeared to be very well separated with t12 samples at negative values on LV_1_ and t26 samples at positive values; the plot of the coefficients showed at positive values the 20 proteins upregulated at t26 and at negative values the 23 proteins downregulated at t26.

The monovariate analysis by Student’s *t* test (*p* < 0.05) identified 20 deregulated proteins in the comparison between controls and 12 days aged meat (t12 vs t26) (Table S1), providing a PLS-DA model including 1 LV, characterized by very good classification performances (accuracy = 100% in fitting and 98.91% in cross-validation; Table S1). Figure 3b reports the score plot of the first LV, with the samples well separated along the y-axis (the solid line represents the delimiter between the two classes); the corresponding coefficients plots (Figure 3b) report 4 proteins as upregulated at t26 (at positive values) and 16 downregulated ones (at negative values).

#### Long-Aging Biomarkers

Kohonen’s SOMs were applied to the late-maturation data set, consisting of 48 samples (24 samples at t0 and 24 at t26) described by 137 variables. The top map (Figure 2c) shows the samples well separated in the two groups, considering its toroidal structure. Also in this case, the three replications of each sample are in the same neuron or in adjacent neurons of the top map. PCA, applied to the weights calculated for each neuron, gave the results reported in Figure S3 (panel c), with neurons containing t0 or t26 samples with a different marker and neurons containing no samples with a black circle. The score plot shows the perfect separation of the neurons containing t0 or t26 samples, the last ones being located at negative scores both on PC_1_ and on PC_2_ and t0 samples located mainly in the opposite position. The corresponding loading plot highlights a group of variables at negative loadings on both PC_1_ and PC_2_, with a larger signal in t26 samples and another group at positive loadings on both PCs with an opposite behavior.

To identify long-maturation biomarkers, PLS-DA was applied with variable selection. The final model included 86 variables and 2 LVs, providing the perfect classification of the samples in fitting and almost perfect results in cross-validation (accuracy = 99.95%, 20% of the samples in the test set at each iteration, 1000 iterations) (Table S1). The model including all the variables showed the best results in cross-validation (20% of the samples in the test set at each iteration, 1000 iterations) with 4 LVs, with an accuracy of 99.5% (100% in fitting): in this case, the results with and without variable selection appear to be quite similar for the cross-validation performances; however, variable selection provides a simpler model (two LVs rather than four), and the risk of overfitting is therefore reduced.

The first two LVs explain 38.10% of the variance of the X variables and 46.27% of class membership (Table S1). The score plot of the first two LVs is reported in Figure 2c: the samples appear to be well separated along the first LV, with the control samples at positive values along LV_1_ and the t26 samples at negative values. The plot of the coefficients (Figure 2c) is separated into two panels for the sake of clarity: 52 variables are upregulated at t26 (positive coefficients), while 34 are downregulated (negative coefficients).

The monovariate approach identified 47 proteins having a different abundance in the comparison between controls and 26 days aged meat (Table S1). The corresponding PLS-DA model contained two LVs and showed performances slightly lower than the multivariate model, with the perfect classification of all the samples in fitting and almost perfect performances in cross-validation (accuracy = 99.64%) (Table S1). The corresponding score and coefficient plots for this model are reported in Figure 3c: the samples are very well separated along LV_1_, with control samples at positive values and t26 values at negative ones. The plot of the coefficients shows 16 markers as upregulated at t26 and 31 markers as downregulated after 26 days.

#### General Biomarkers of Aging

Kohonen’s SOMs were applied to the general-aging data set, comprising all the 69 samples (24 samples at t0, and 21 and 24 samples at t12 and t26, respectively) described by 137 variables. The neurons in the top map containing samples of different groups are indicated by a different color (Figure 2d): if t0 vs all the other samples are considered, the samples appear to be well separated on the top map, considering the toroidal structure, while the separation is not so clear if the three distinct groups are considered. Again, the three replications of each sample are in the same neuron or in adjacent neurons of the top map. PCA, applied to the weights calculated for each neuron, gave the results reported in Figure S3 (panel d), with neurons containing t0, t12, or t26 samples with different markers and neurons containing no samples with a black circle. The score plot shows quite a good separation of the neurons containing t0 samples with respect to neurons containing the other two groups of samples: t0 samples are mainly located at positive scores on PC_1_ and at negative ones on PC_2_, while t12 and t26 neurons are located in the opposite position. The corresponding loading plot highlights a group of variables at negative loadings on PC_1_ and at positive ones on PC_2_, with a larger signal particularly in t26 samples and partially in t12 samples with respect to t0 samples, while the variables located in the opposite behavior show a larger signal in t0 samples.

To identify general biomarkers of aging, PLS-DA was applied to the overall data set comparing t0 samples vs all other samples with variables selection. The final model included 97 variables and 6 LVs, providing the perfect classification of the samples in fitting and almost perfect results in cross-validation (accuracy = 99.82%) (Table S1). The comparison with results obtained without variable selection proved the effectiveness of the selection procedure in providing the most discriminating biomarkers, achieving in the meantime a simpler model with higher performances in cross-validation and reducing the risk of overfitting. The model including all the variables in facts showed the best results in cross-validation (20% of the samples in the test set at each iteration, 1000 iterations) with 5 LVs, with an accuracy of 98% (100% in fitting).

The first two LVs explain 30.48% of the variance of the X variables and 38.76% of class membership (Table S1). Figure 2d shows the score plot of the first two LVs, with samples well separated in the space given by the first two LVs; the plot of the coefficients (Figure 2d) is separated in two panels for more clarity: 56 variables are upregulated in t12 and t26 samples (positive coefficients), while 41 are downregulated (negative values).

The monovariate approach identified 32 proteins with different abundances in the comparison between controls and other samples (Table S1). The corresponding PLS-DA model contained six LVs and showed performances lower than the multivariate model, with the perfect classification of all the samples in fitting and good performances in cross-validation (accuracy = 95.39%) (Table S1). The corresponding score and coefficient plots for this model are reported in Figure 3d: the samples are quite well separated along LV_1_, with t0 samples at negative values and t12 and t26 values at more positive ones. The plot of the coefficients shows 10 markers as upregulated with aging and 22 markers as downregulated with aging.

**Figure 4:**
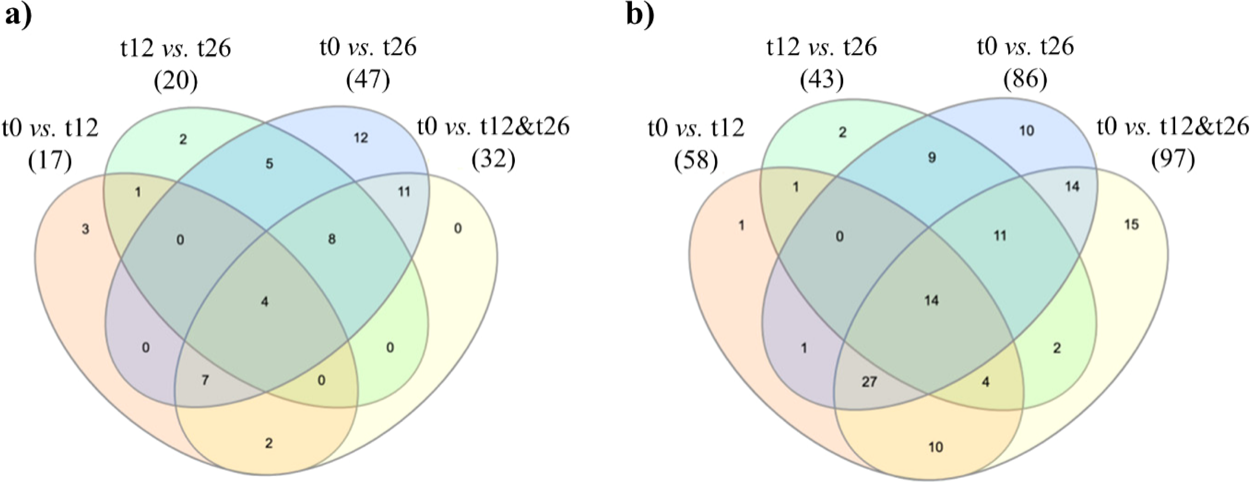
Venn diagrams showing the number of significantly affected proteins by early, late, long and general aging. Significant proteins were selected based on (a) univariate (Student’s t test) or (b) multivariate (PLS-DA) analysis (see Table S1 for gene/protein names).

The results obtained by the different approaches (fold-change, p-level calculated by the monovariate approach, coefficient of the PLS-DA model, and loadings of the first two PCs calculated from the Kohonen network) are reported in Table S1 and Figure S3, showing the general agreement between the different approaches, notwithstanding the more exhaustiveness reached by PLS-DA. The present results showed that the multivariate statistical analysis was more comprehensive and, for all the considered comparisons, allowed us to identify more than double of biomarkers detected by univariate analysis. Overlapping and nonoverlapping proteins with changes of the abundance after 12 and 26 days of aging, detected by univariate and multivariate analysis, are reported in Figure 4 (for protein names, see Table S1). Hemoglobin alpha and beta subunits and carbonic anhydrase 3 were the most decreased proteins in the LD muscle after 12 days of aging. Mimecan, chains of collagen alpha-1(I), and alpha-2(I) were instead identified as the most decreased proteins in meat aged for 26 days. Mimecan expression is associated with collagen deposition, while post-mortem degradation of collagen fibers plays a key role in meat tenderness by altering the connective tissue structure.^33^ Therefore, the greater tenderness of meat after 26 days of maturation can be ascribed to degradation of connective tissue, a process that does not seem to be involved during the early aging, as suggested by the absence of chains of collagen.

#### Functional Enrichment, Pathway, and Protein–Protein Interaction Network Analyses

**Figure 5:**
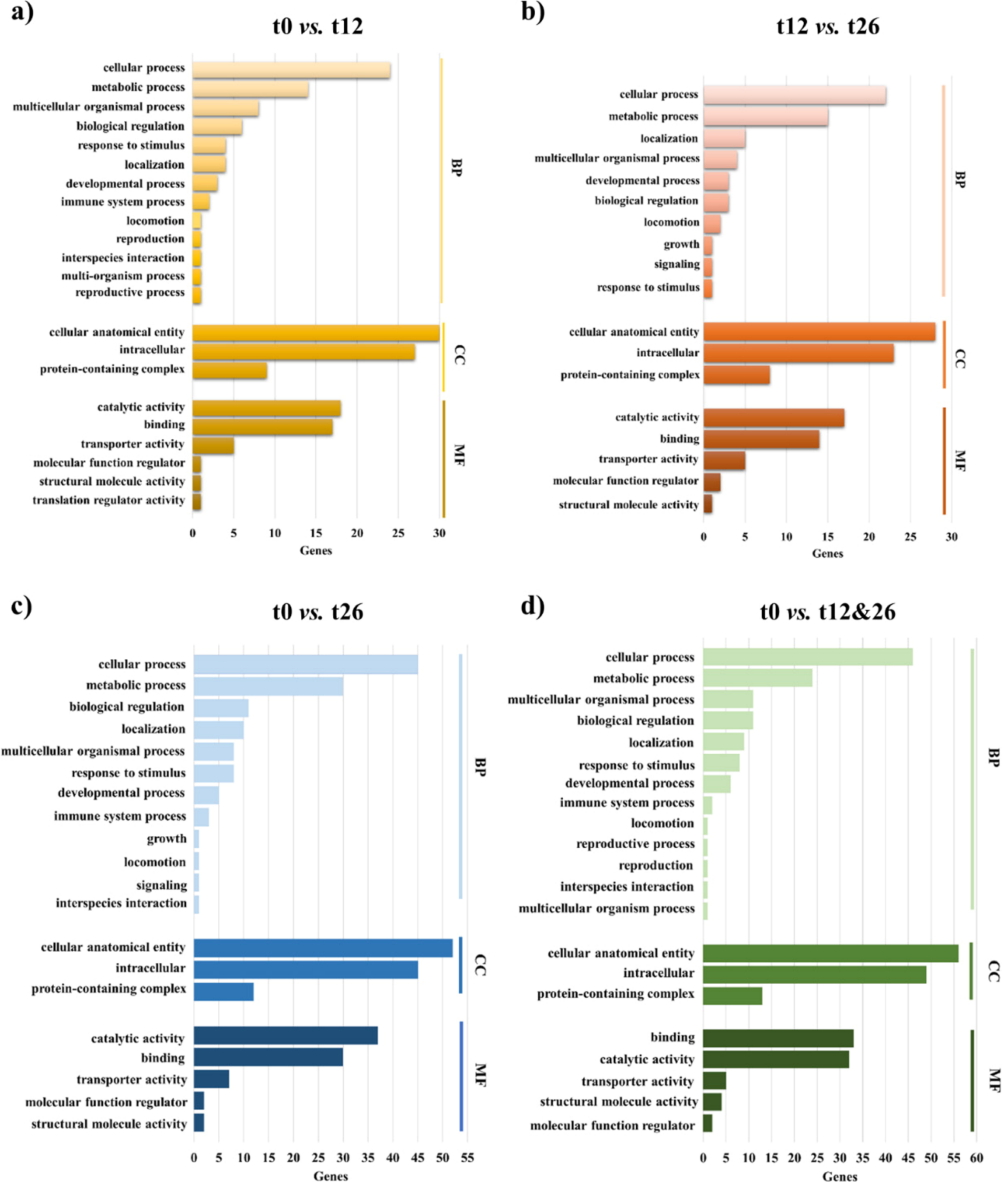
Functional classifications of biomarkers of meat tenderness. GO analysis of proteins followed by BP, CC and MF, depicts the functional distribution of proteins in bovine meat after (a) early, (b) late, (c) long, and (d) general aging. The number of assigned genes may be greater than the number of recognized genes as the same gene can be included in different categories.

Proteins detected by multivariate analysis (PLS-DA) were subjected to bioinformatics to explore the biological functions and pathways and to construct specific molecular networks (Table S1). In order to identify overrepresented biological processes (BP), cellular components (CC), and molecular functions (MF), proteins were subjected to a GO enrichment analysis using the PANTHER classification system (Figure 5). The main enriched categories were similar for the different comparisons carried out, that is, cellular and metabolic processes (for the BP category), cellular anatomical entity, intracellular and protein complex (for the CC category), binding, catalytic, and transporter activity (for MF), with a higher percentage of the identified genes in long and general aging. To determine key proteins in the function networks, the STRING online tool was used to analyze protein–protein interactions. The analysis resulted in statistically significant networks (shown in Figure S4) with an average node degree (i.e., number of interactions at the score threshold that a protein has on the average in the network) of 1.42 (t0 vs t12), 0.78 (t12 vs t26), 1.98 (t0 vs t26), and 2.35 (t0 vs t12&26). This pointed out the presence of higher connected neighborhoods in the network after 26 days of aging, as indicated by the presence of larger groups of interacting proteins. The clusters determined by the k-means method were found to be enriched in interacting proteins of ″muscle structure and contraction″ and ″energy metabolism″ in all the comparisons and also in proteins of ″response to stress and to oxidative stress″, particularly after the long and general aging. Lastly, KEGG pathway enrichment was performed to extract the biological pathways related to the differentially abundant proteins (Table S1). The most significant enriched pathway was ″muscle contraction″ (*q*-value = 5.83e-06) for early aging; ″glycolysis/gluconeogenesis″ and ″carbon metabolism″ (both with *q*-value = 2.48e-07) for late aging; and ″carbon metabolism″ for long and general aging (*q*-value 2.41e-13 and 7.86e-10, respectively). This analysis revealed that dysregulated proteins of early maturation were highly associated with pathways related to muscle structure and contraction (i.e., ″cardiac muscle contraction″, ″hypertrophic cardiomyopathy″, and ″dilated cardiomyopathy pathways″); those of late aging were also involved in ″biosynthesis of amino acids″, ″oxidative phosphorylation″, and in metabolism of carbohydrates (galactose, starch, and sucrose), while proteins with altered abundance after long aging were also implicated in ″metabolism of glyoxylate, dicarboxylate, and propanoate.″

In summary, the proteome changes in the LD muscle of Charolais cattle after 12 or 26 days of aging were investigated herein. The gel-free high throughput proteomics approach coupled to advanced statistics confirmed 24 out of the 39 unique proteins previously identified^2^ and allowed us to identify a huge amount of other additional biomarkers, which were not previously detected by gel-based proteomics analysis. In conclusion, the data gathered by this study enlarge the panel of biomarkers associated with meat tenderness of Charolais cattle^34−36^ and provide a powerful method based on gel-free proteomics and multivariate chemometric techniques that can be applied also in other areas of meat science.

## Supporting Information Available

Experimental workflow and overview of the gel-free proteomic, statistical, and bioinformatic analyses; results of PCA; protein–protein interaction networks (PDF)
Univariate and multivariate statistical analysis data (Excel)
Warner–Bratzler shear (WBS) test results; data on odor, taste, flavor, juiciness, sweet, tenderness, saltiness, bitterness, acidity, chewiness, and overall acceptability (Excel)
Results obtained for the mono- and multi- variate models (PDF)
Percentage of variance explained by each latent variable on X and Y variables for each comparison carried out (PDF)
Percent of gene hit against the total number of genes and percent of gene hit against the total; IDs and labels of matching proteins in the network for KEGG t0 vs t12, KEGG t12 vs t26, KEGG t0 vs t26, and KEGG t0 vs t12&t26 (Excel)
Samples, univariate and multivariate statistical analysis data; correlation matrix; and percentage of variance of LVs (Excel)
