# Supplementary material for: Kohonen Artificial Neural Network and Multivariate Analysis in the Identification of Proteome Changes during Early and Long Aging of Bovine Longissimus dorsi Muscle Using SWATH Mass Spectrometry
Source: J Agric Food Chem. 2021 Sep 15;69(38):11512–22. doi: 10.1021/acs.jafc.1c03578 (PMC8485349; doi:10.1021/acs.jafc.1c03578)

Supplementary Figure 1

Experimental workflow and overview of the gel-free proteomics, statistical and bioinformatics analyses. The figure was created with BioRender.com.

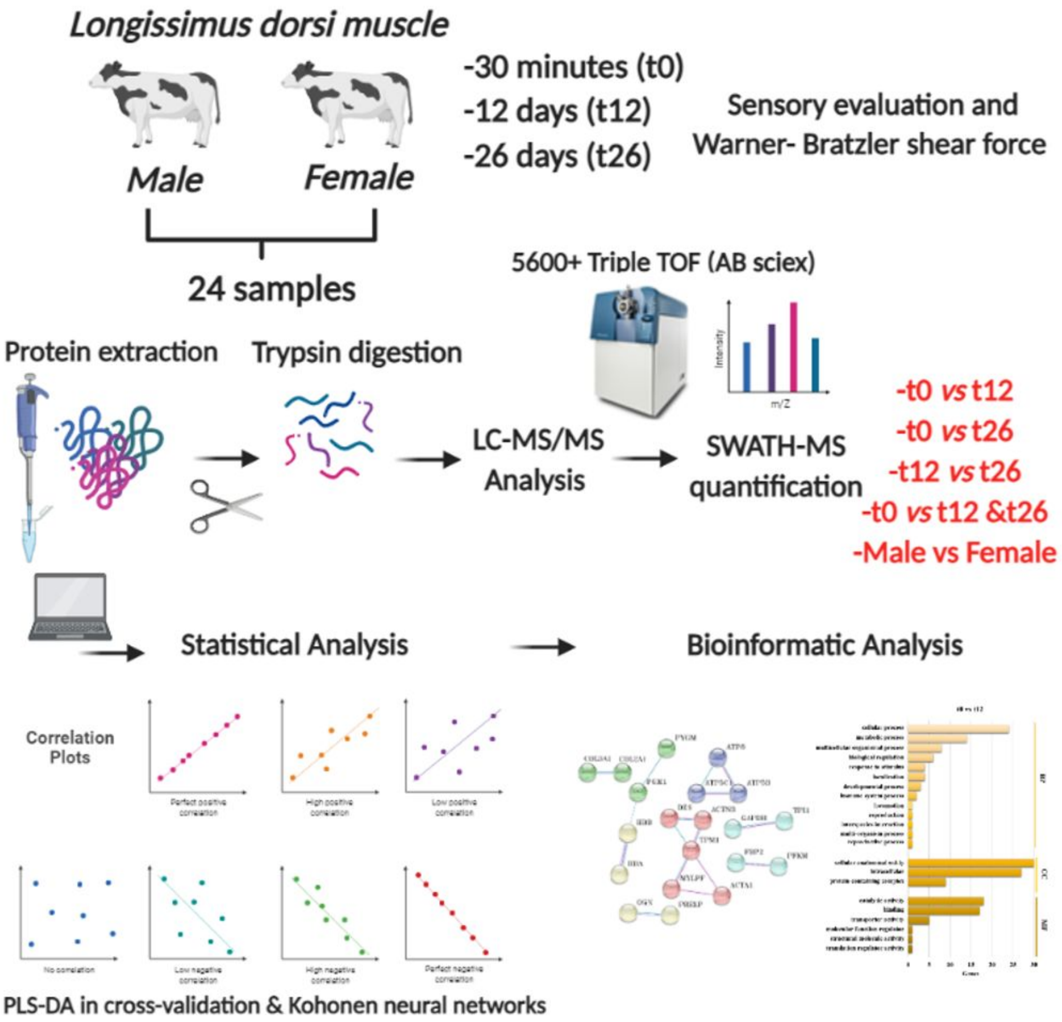

Supplementary Figure 2

Results of PCA carried out on the overall dataset consisting of 69 samples (24 t0 samples, 21 t12 samples and 24 t26 samples) described by 138 variables (protein signals): score plot of the first two PCs with the samples labelled according to **(a)** the gender and **(b)** the time of maturation; score plot of the PC3 and PC4 with the samples labelled according to **(c)** the gender and **(d)** the time of maturation; loading plot of **(e)** the first two PCs and of **(f)** PC3 and PC4.

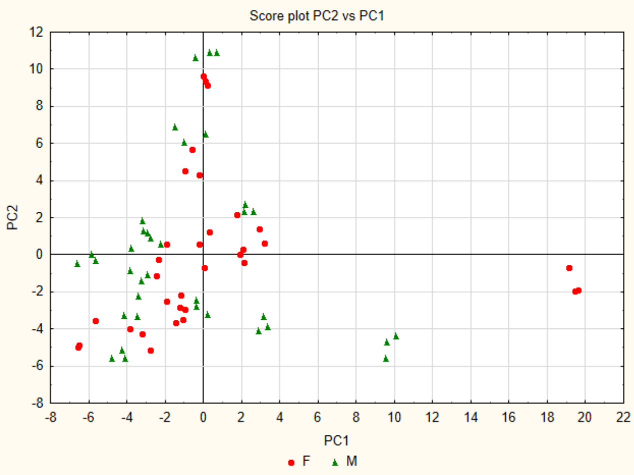

(a)

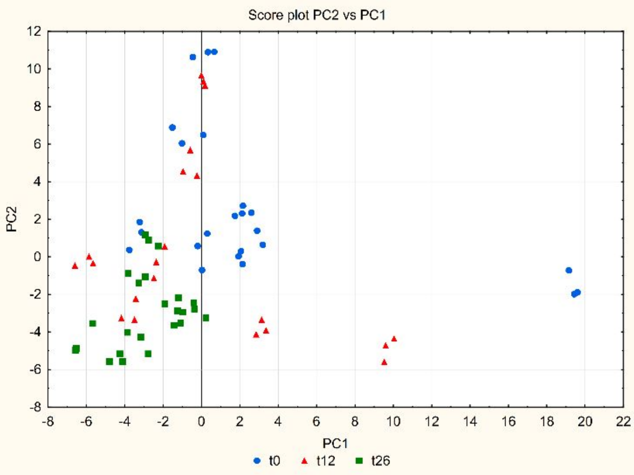

(b)

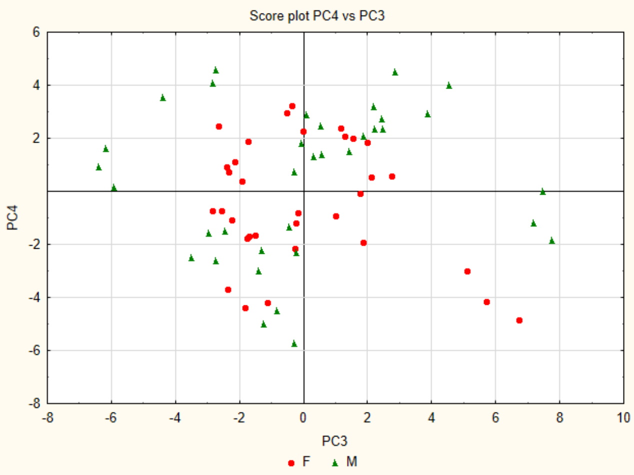

(c)

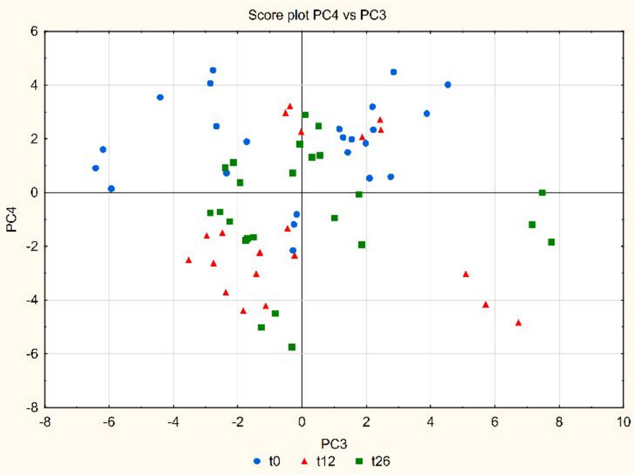

(d)

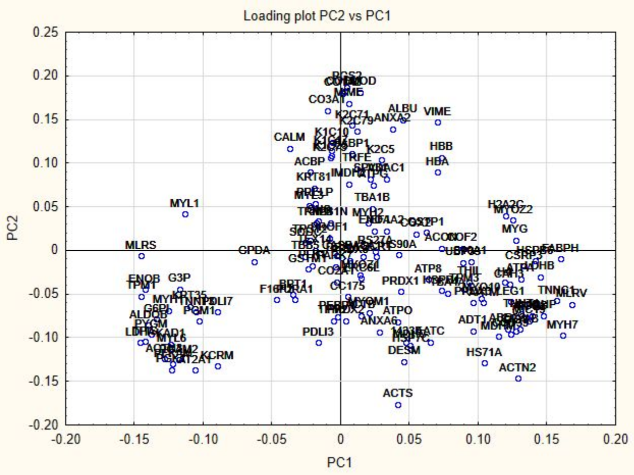

(e)

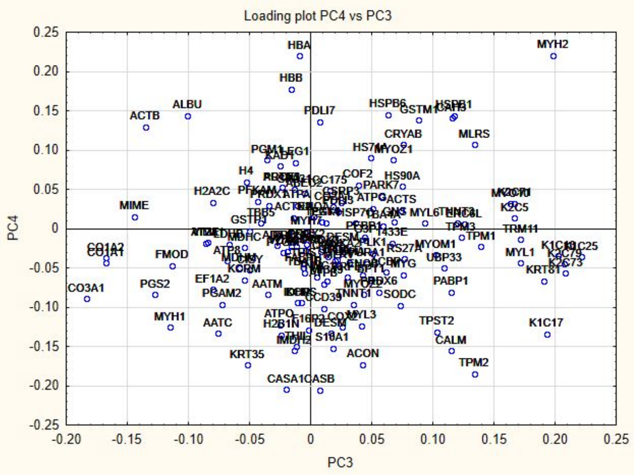

(f)

Supplementary Figure 3

Results of PCA carried out on the weights of the neurons of the Kohonen's top map; score plot and loading plot of the first two PCs for: early **(a)**, late **(b)**, long **(c)**, general **(d)** aging. Neurons in the score plot are labelled according to the class of samples they contain; if the neurons do not contain samples, they are indicated as black circles.

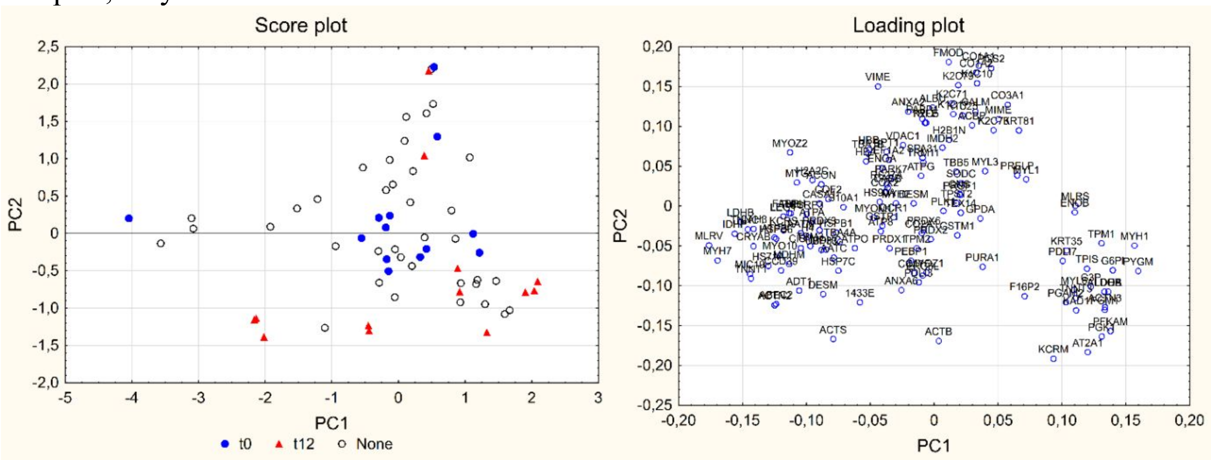

(a)

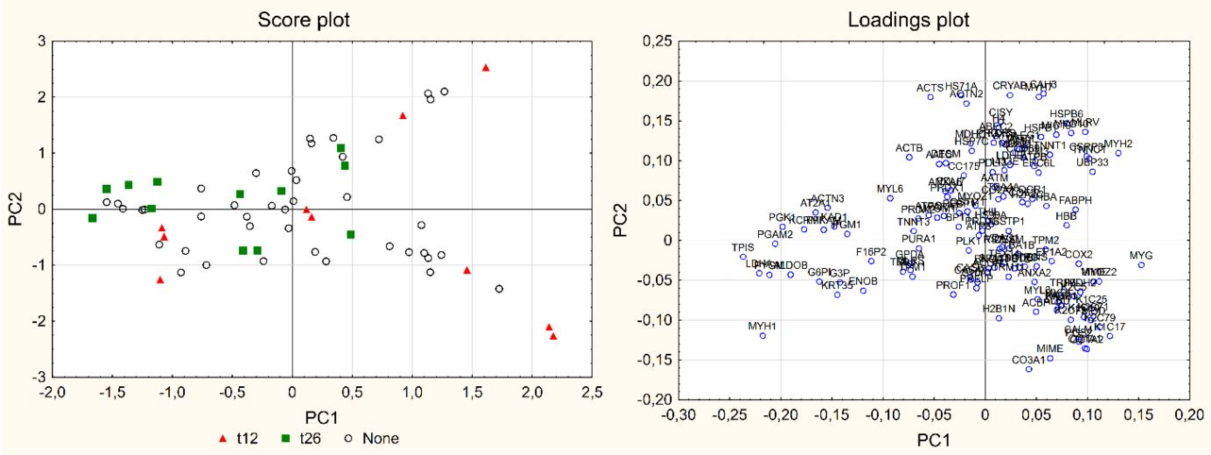

(b)

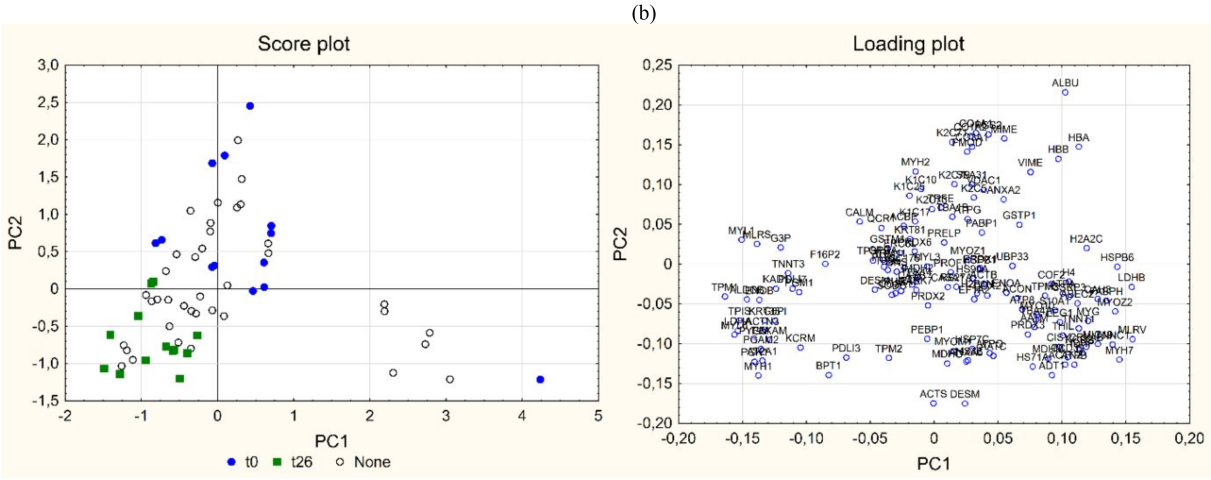

(c)

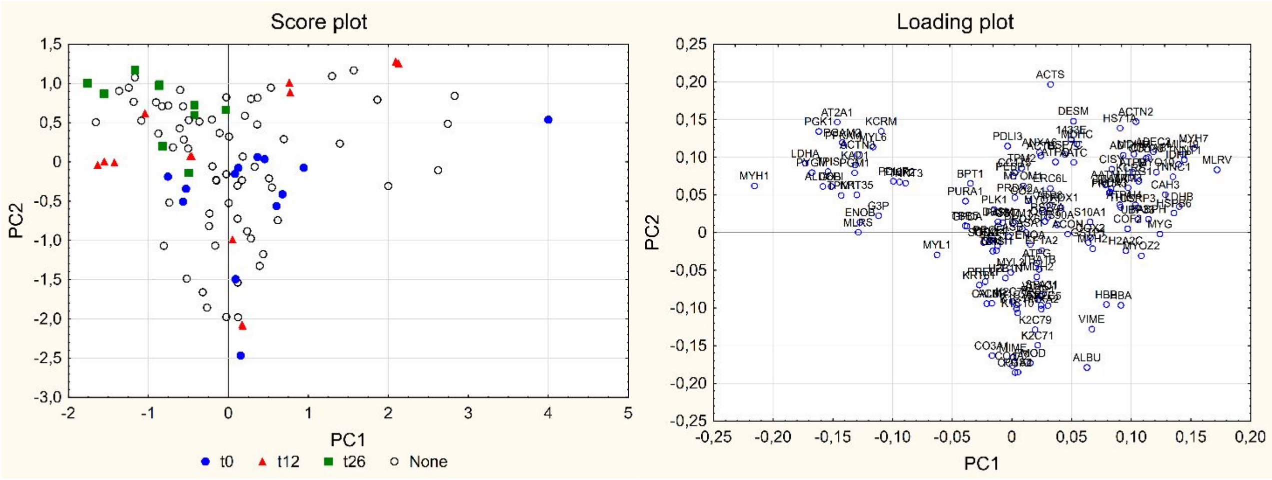
Score plot
PC1
PC2
t0
t12
t26
None
Loading plot
PC1
PC2
MYH1
ACTS
DESM
ACTN2
HS71A
AT2A1
PGK1
KCRM
LDHA
MYH7
MLRV
CAH3
MYG
PURA1
BPT1
MYL1
ENOB
G3P
K2C79
K2C71
VIME
ALBU
HBB
HBA
CO3A1
S10A1

(d)

Supplementary Figure 4

The protein-protein interaction networks as analysed by String software for proteins having altered abundance after (a) early, (b) late, (c) long, and (d) general ageing. A pink line indicates experimental evidence, while a light blue line indicates database evidence.

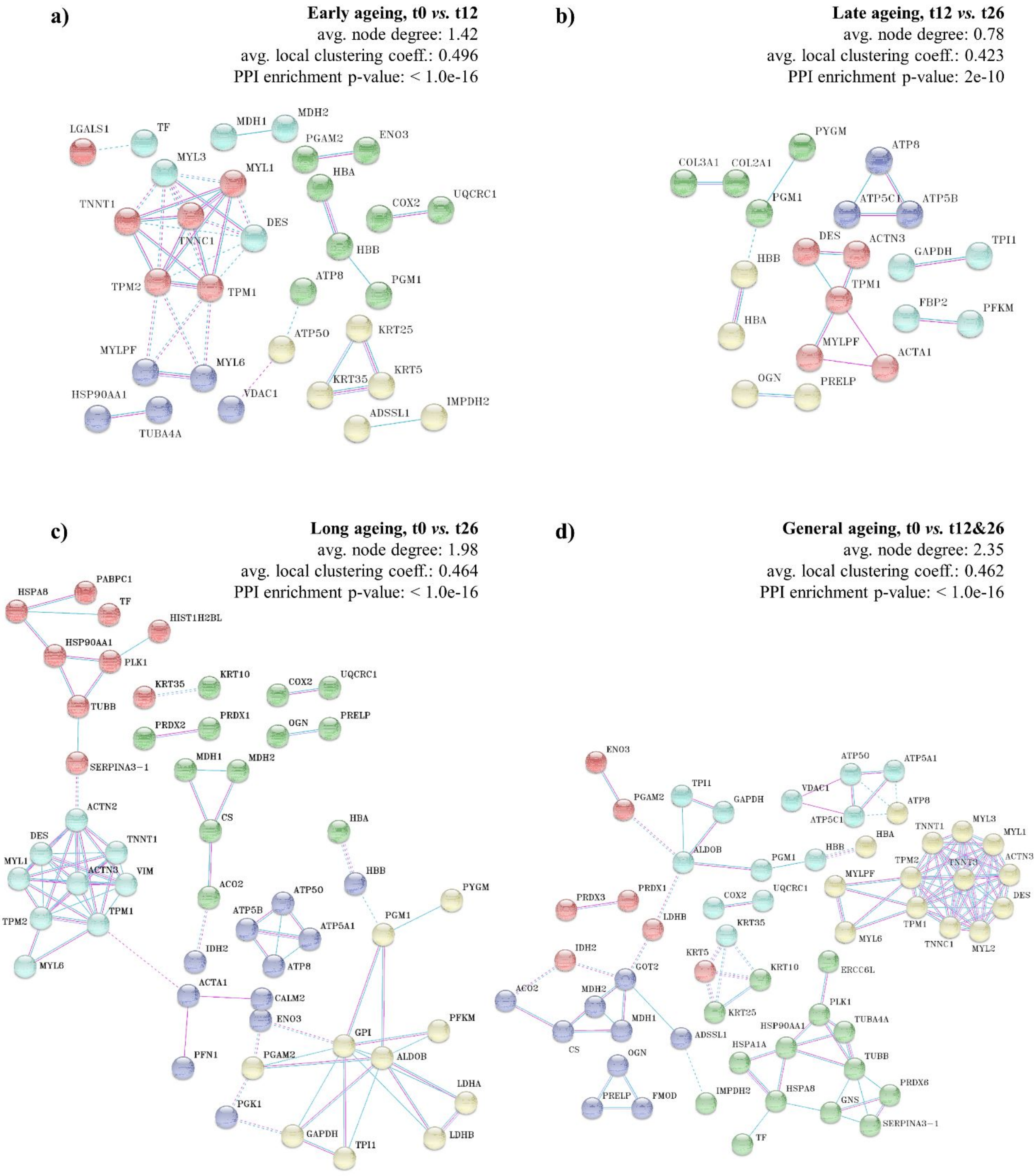

Supplement: Supplementary file 1 — jf1c03578_si_001.pdf [file jf1c03578_si_001.pdf]
