# Supplementary material for: Kohonen Artificial Neural Network and Multivariate Analysis in the Identification of Proteome Changes during Early and Long Aging of Bovine Longissimus dorsi Muscle Using SWATH Mass Spectrometry
Source: J Agric Food Chem. 2021 Sep 15;69(38):11512–22. doi: 10.1021/acs.jafc.1c03578 (PMC8485349; doi:10.1021/acs.jafc.1c03578)
Supplement: Supplementary file 4 — jf1c03578_si_004.pdf [file jf1c03578_si_004.pdf]

Supplementary Table 2:
Results obtained for the mono- and multi- variate models calculated. For each comparison, several indexes are given: number of original variables (N° variables) and number of latent variables included in the model (N° LVs), Accuracy% (Acc%), NER%; Sensitivity %, Specificity % and Precision % for each class. All the results are given in fitting and in cross-validation (CV) (random selection of 5 cancellation groups; 1000 iterations).

		N° Variables	N° LVs	Acc %	NER %	Sensitivity %		Specificity %		Precision %	
						Control	Other class	Control	Other class	Control	Other class
**t0 *vs.* t12**	**Fitting**	17	1	95.56	95.83	91.67	100	100	91.67	100	91.30
**monovariate test**	**CV**			95.39	95.64	92.41	98.87	98.87	92.41	98.96	91.77
**t0 *vs.* t12**	**Fitting**	58	2	100	100	100	100	100	100	100	100
**multivariate test**	**CV**			99.77	99.77	99.62	99.93	99.93	99.62	99.94	99.58
**t12 *vs.* t26**	**Fitting**	20	1	100	100	100	100	100	100	100	100
**monovariate test**	**CV**			98.91	98.97	99.98	97.97	97.97	99.98	97.75	99.98
**t12 *vs.* t26**	**Fitting**	43	2	100	100	100	100	100	100	100	100
**multivariate test**	**CV**			99.98	99.98	99.98	99.98	99.98	99.98	99.98	99.98
**t0 *vs.* t26**	**Fitting**	47	2	100	100	100	100	100	100	100	100
**monovariate test**	**CV**			99.64	99.64	99.72	99.56	99.56	99.72	99.56	99.72
**t0 *vs.* t26**	**Fitting**	86	2	100	100	100	100	100	100	100	100
**multivariate test**	**CV**			99.95	99.95	100	99.90	99.90	100	99.90	100
**t0 *vs.* t12&t26**	**Fitting**	32	6	100	100	100	100	100	100	100	100
**monovariate test**	**CV**			95.39	96.48	92.98	99.98	92.98	99.98	88.18	99.99
**t0 *vs.* t12&t26**	**Fitting**	97	6	100	100	100	100	100	100	100	100
**multivariate test**	**CV**			99.82	99.85	99.96	99.75	99.75	99.96	99.53	99.98
