# Supplementary material for: Kohonen Artificial Neural Network and Multivariate Analysis in the Identification of Proteome Changes during Early and Long Aging of Bovine Longissimus dorsi Muscle Using SWATH Mass Spectrometry
Source: J Agric Food Chem. 2021 Sep 15;69(38):11512–22. doi: 10.1021/acs.jafc.1c03578 (PMC8485349; doi:10.1021/acs.jafc.1c03578)
Supplement: Supplementary file 5 — jf1c03578_si_005.pdf [file jf1c03578_si_005.pdf]

**Supplemental Table 2:** % of variance explained by each latent variable on X and Y variables for each comparison carried out.

| Variables selected by multivariate analysis | | | | Variables selected by uniivariate analysis | | | |
|---|---|---|---|---|---|---|---|
| | | X variables | Y variable | | | X variables | Y variable |
| | | %Expl. Var. | %Expl. Var. | | | %Expl. Var. | %Expl. Var. |
| EARLY | LV1 | 15,46 | 40,14 | EARLY | LV1 | 32,68 | 32,89 |
| | LV2 | 18,89 | 5,60 | | LV2 | 9,73 | 6,30 |
| | LV3 | 9,29 | 1,88 | | LV3 | 9,41 | 1,71 |
| | LV4 | 6,84 | 0,78 | | LV4 | 6,12 | 1,69 |
| | LV5 | 3,46 | 0,57 | | LV5 | 7,84 | 0,93 |
| | LV6 | 5,54 | 0,16 | | LV6 | 4,47 | 1,26 |
| | LV7 | 3,85 | 0,15 | | LV7 | 3,44 | 0,92 |
| | LV8 | 3,58 | 0,09 | | LV8 | 6,83 | 0,17 |
| | LV9 | 2,14 | 0,12 | | LV9 | 5,01 | 0,11 |
| | LV10 | 2,38 | 0,07 | | LV10 | 3,63 | 0,06 |
| LATE | LV1 | 18,94 | 41,82 | LATE | LV1 | 32,17 | 34,49 |
| | LV2 | 9,01 | 4,43 | | LV2 | 11,42 | 5,51 |
| | LV3 | 9,11 | 0,70 | | LV3 | 9,31 | 2,28 |
| | LV4 | 8,32 | 0,59 | | LV4 | 4,60 | 1,10 |
| | LV5 | 5,75 | 0,46 | | LV5 | 6,87 | 0,28 |
| | LV6 | 5,22 | 0,25 | | LV6 | 6,97 | 0,17 |
| | LV7 | 3,57 | 0,23 | | LV7 | 3,61 | 0,14 |
| | LV8 | 4,00 | 0,15 | | LV8 | 2,85 | 0,12 |
| | LV9 | 3,01 | 0,12 | | LV9 | 3,54 | 0,07 |
| | LV10 | 2,05 | 0,12 | | LV10 | 2,00 | 0,11 |
| LONG | LV1 | 24,58 | 39,63 | LONG | LV1 | 41,17 | 37,81 |
| | LV2 | 13,52 | 6,65 | | LV2 | 6,41 | 7,14 |
| | LV3 | 6,88 | 1,85 | | LV3 | 6,92 | 1,77 |
| | LV4 | 3,39 | 1,01 | | LV4 | 7,10 | 0,85 |
| | LV5 | 3,19 | 0,43 | | LV5 | 8,09 | 0,52 |
| | LV6 | 3,97 | 0,15 | | LV6 | 2,88 | 0,62 |
| | LV7 | 3,62 | 0,08 | | LV7 | 2,03 | 0,45 |
| | LV8 | 2,92 | 0,06 | | LV8 | 1,67 | 0,29 |
| | LV9 | 2,38 | 0,04 | | LV9 | 1,91 | 0,16 |
| | LV10 | 1,70 | 0,05 | | LV10 | 2,04 | 0,08 |
| GENERAL | LV1 | 17,38 | 29,16 | GENERAL | LV1 | 35,63 | 27,53 |
| | LV2 | 13,10 | 9,60 | | LV2 | 8,02 | 7,70 |
| | LV3 | 7,08 | 4,05 | | LV3 | 10,47 | 2,79 |
| | LV4 | 4,23 | 1,42 | | LV4 | 7,44 | 2,07 |
| | LV5 | 4,37 | 0,33 | | LV5 | 5,68 | 1,00 |
| | LV6 | 3,27 | 0,24 | | LV6 | 3,65 | 0,53 |
| | LV7 | 2,79 | 0,16 | | LV7 | 3,34 | 0,26 |
| | LV8 | 2,00 | 0,12 | | LV8 | 2,40 | 0,32 |
| | LV9 | 1,97 | 0,07 | | LV9 | 2,07 | 0,25 |
| | LV10 | 2,14 | 0,05 | | LV10 | 2,54 | 0,08 |
